# Evolving Healthcare Delivery in Neurology During the Coronavirus Disease 2019 (COVID-19) Pandemic

**DOI:** 10.3389/fneur.2020.00578

**Published:** 2020-05-29

**Authors:** Patrick M. Chen, Thomas M. Hemmen

**Affiliations:** Department of Neurosciences, University of California, San Diego, San Diego, CA, United States

**Keywords:** COVID-19, pandemic, general neurology, healthcare workflow, stroke, PPE, telemedicine, burnout

## Introduction

Coronavirus Disease 2019 (COVID-19) is caused by the acute respiratory syndrome coronavirus 2 (SARS-CoV-2) and has led to the development of a rapidly evolving pandemic (1). The pandemic changed the assumptions made by most developed health care system: ample supplies and an overwhelmingly safe environment for patients and healthcare providers. Hospital resources and supply are no longer secure, and the potential risk to patients and caregivers is increased. As neurologists, we face these challenges in many areas.

Here, we discuss the impact of the pandemic on neurology work flow in four areas: inpatient care, outpatient care, research, and ethics.

## Inpatient Management

One key lesson from the COVID-19 experience internationally is the rapid depletion and scarcity of medical supplies [e.g., personal protective equipment (PPE) and mechanical ventilators], beds, and staff—an increasing occupational hazard for health care workers (2–4). We must critically evaluate our workflow and resource utilization in this crisis. Acute stroke alerts present the most direct potential interface with COVID-19 patients. Existing stroke alert paradigms focus on high sensitivity for stroke detection with generally low specificity, requiring high resource utilization (5).

Several new workflows and consensus statements have been proposed for “protected” stroke alerts (6–9). Overarching themes include expanded pre-screening in peri-hospital setting, widespread PPE training, designated “safety leaders” for monitoring proper precautions, limited examinations, and telemedicine. Similarly, we have demonstrated the practicality of implementing tele-stroke video technology in the emergency room for initial triage during the pandemic (10). Rapidly implementing a large-scale “protected code” policy requires multidisciplinary coordination with hospital administration, other subspecialties (e.g., emergency department), and frequent feedback on the policies effectiveness from the frontline (e.g., nursing, ancillary staff, and trainees). In the future, the stroke alert could consolidate other COVID-19-related tests, such as chest imaging. How these protected workflow trends will affect time metrics and stroke care outcomes is yet to be determined.

Neurologic admissions and transfers to the hospital must be triaged and prioritized. We previously had the luxury of prolonged observation and extended outpatient workups, but we must now consider the exposure risks of prolonged hospitalization. Surgical specialties have significantly reduced “elective” surgery (3). In a similar vein, we should be judicious in determining if the benefits of admission or intervention supersede the potential dangers and resource utilization in the current crisis. We often call upon the neurological intensive care unit (ICU) for co-management, though these beds and staff are also needed for COVID-19 overflow. In a pandemic, it is reasonable to reserve resources, such as thrombectomy, to patients that would benefit the most, according to high-level (Class 1, Level A Evidence) guidelines (7). Ideally separate units should be used to isolate neurologic patients with COVID-19 from neurology patients without the disease.

Beyond stroke patients, neurologists interface with the COVID-19 population for symptoms including anosmia, encephalopathy, headache, or meningitis-encephalitis rule out. We must be cautious in pigeonholing a COVID-19 patient and must resist substituting proxy diagnostics for a clinical exam because of infection risk. Ancillary testing (e.g., EEG and CT scans) involve not only the machinery that will need to be disinfected but also personnel with risks for viral exposure. Yet, standard of care, if indicated, should not be withheld due to COVID-19. Given variability in individuals risk tolerance, a unified protocol may help remove these possible diagnostic biases in COVID-19 patients. Finally, with a need for mechanical ventilators and ICU resources, our teams will need to be practical but still thorough in prognostication of catastrophic neurologic disease to assist resource allocation.

Many institutions share similar policies to reduce COVID-19 transmission (9). At our institution, family visits are restricted, and all admitted patients receive a SARS-COV2 PCR test. Regarding PPE, aerosolized high-risk patients require N95 masks/powered air purifiers (PAPR) with eye protection, gowns, and gloves, while other inpatients require surgical masks, gloves, and eye protection (6). In circumstances of limited history, such as stroke codes or persons under investigation, an abundance of care should be taken. The possibility of asymptomatic COVID-19 carriers or occult history should be considered in our patients and consults, underpinning the importance of universal precautions and rapid COVID-19 testing when available. Team members at high risk (e.g., immunosuppression and those over age 60) are triaged to avoid direct contact (e.g., telemedicine role) when possible. Finally, should a team member be exposed to COVID-19 or show concerning symptoms, we follow the institutions policy regarding symptom monitoring, self-quarantine, and testing.

The day-to-day routines of neurologists in the hospital have changed. For our institution, rounds have been streamlined to one senior team member, and team rounds are carried out over video conferencing. We practice six feet of distance amongst staff and patients and consider telephone-video conversation when possible except for critical physical examinations. We perform limited, but practical, neurologic examinations (at minimum: mental status, cranial nerves, and gross motor skills) focused on localization that guides changes in management. COVID-19-positive or PUI patients are seen last to reduce transmission. Neurologists have the challenge of protecting the specialties tenants of diagnostic exactness and personalized patient rapport despite these limitations.

Finally, we have yet to see the long-term effects of COVID-19 on trainee education and mental health. The Accreditation Committee of General Medical Education have made new exceptions to previous training requirements considering the pandemic, though there is concern this may lead to suboptimal learning conditions. Currently, neurology trainees may be deployed to non-specialty services while primary teams are downsized. Didactics are converted to video conferences, clinics are conducted via telemedicine, and the tradition of neurology bedside rounds and examination are curtailed. Do these adaptations add to or deprive neurology training, and will these changes persist after the pandemic? Similarly little is known about the impact of COVID-19 on the psychological health of our team members who face a number of stresses: occupational risk, evolving policy changes, and unprecedented ethical decisions. The risk for trainee burnout—occupational, mental, emotional, and physical exhaustion—is high. A prophylactic solution to this by leadership should take the form of self-care initiatives, multidisciplinary mental health support groups, and frequent open forums (e.g., town halls) for trainees and all team members.

## Outpatient Care and Telemedicine

A substantial portion of the neurologic population is classified by the Center for Disease Control (CDC) as “high risk” (e.g., elderly, neuromuscular, immunosuppressed) for COVID-19 illness (11, 12). How can we best protect this vulnerable population while providing continuity of care?

A review of current literature shows various subspecialties—multiple sclerosis (13), vascular (8), neuromuscular (14), and epilepsy (15)—have attempted to tackle this question in the form of consensus statements by subspecialty leaders. Recommendations are broad but share consistent themes: (1) screen all patients and use universal precautions in clinic visits; (2) prevent unnecessary medical facility visits; (3) triage diagnostic workups; (4) develop individualized contingency plans; and (5) avoid drastic regimen changes based on speculative links between COVID-19 and neurologic disease.

The use of telemedicine platforms is critical when providing care to high-risk populations. Pre-pandemic literature suggested telehealth was not inferior to face-to-face clinic visits for outcomes across neurologic subspecialties (16). The expansion of Medicare coverage beyond rural areas and relaxing tele-HIPAA requirements in response to the pandemic (17, 18) has catalyzed rapid and wide implementation. The technology is versatile and could be expanded to monitoring with remote devices (e.g., accelerometers in Parkinson's disease), neuro-rehabilitation, and providing a hotline to curb isolation in the elderly and disabled. Proponents of telemedicine highlight its role in the “4 Cs”: better access to care, greater convenience, enhanced patient comfort, and better confidentiality. There is also an added new C—“contagion” (19). Telemedicine is limited in the funduscopic, neuromuscular, and vestibular exams, and there remain concerns regarding consistent technology access and consistent privacy standards. We urge neurologist to address previous methodological flaws in the literature through collection of outcomes with neuro-telehealth. By addressing past infrastructure gaps, we may develop a feasible telehealth system for a high-quality standard of care post-pandemic. This data will help establish the marginal benefits of in-person visits over tele-visits. In many situations, this benefit may be much smaller from a risk–benefit and cost analysis standpoint than traditionally thought.

A key question remains of how we will prepare for the return of neurologic patients with delayed diagnosis because of COVID-19. The number of stroke and myocardial disease hospital presentations decreased during the peak of the pandemic (20). These patients avoided and delayed health care due to isolation and quarantine, and this is likely applicable to other chronic neurologic conditions. As neurologists, we will need to explore the effects of isolation and fear on the outcomes of our neurologic patients. It is our responsibility to be proactive in educating our patients on the urgency of evaluation when appropriate, perhaps with more frequent tele-health follow-up, designated post-hospitalization follow-up coordinators, and large public organizational campaigns (e.g., Stroke F.A.S.T campaign). We expect to see an upsurge in delayed neurologic complications as pandemic restrictions lighten, which may further exacerbate healthcare resource limitations.

## COVID-19 and Neurology Research

The pandemic has created a fervor within the research community, and neurology is not an exception. A number of small, observational retrospective studies have emerged with reports of Guillain-Barre (21) syndrome, hemorrhagic encephalopathy (22), and stroke (23). There is speculation that anosmia may be from olfactory involvement of SARS-CoV2 (24). Yet, it remains unclear if these reported correlations also lend to causation. Editorial boards have pushed these findings to the forefront by offering pre-review releases, expedited review, and open access. While rapid information dissemination is important in uncertain times, we caution against the risk of “research exceptionalism” (25). As the pandemic matures, the mentality of “better than nothing” should be transitioned to similar rigorous pre-pandemic publication standards if the findings are to be of clinical meaning. Pandemic opportunism should not compromise the past standard of research integrity. Given this, we must be cautious in how we interpret findings, especially when considering diverging from pre-pandemic standard of care.

COVID-19 has posed many challenges to ongoing large clinical trials. Quarantine and travel restrictions have forced the pause of enrollment and rigid study protocols place several logistical strains on research staff. Nevertheless, there remains a moral obligation to current study participants to complete these studies. How this is handled is complex and individualized by the study group. As the pandemic recedes, the impact of the pandemic directly (e.g., loss of participants or data) and indirectly (e.g., infection as a confounder) will need to be accounted for in result analysis and explored further.

Ultimately, we must leverage our research focus and resources wisely. The societal drive to understand COVID-19 should not also come at the expense of our non-COVID-19 neurologic patients. While the neurologic complications have captured the public eye, we should consider questions around quality improvement, personnel wellness, and the impact of the aforementioned workflow changes. An important task moving forward is to be methodical in our collection of data for COVID-19 neurologic patients if we are the truly understand its role in the central nervous system. This will likely take the form of multi-center consortiums with a standardized protocol to create large prospective databases.

## Ethics and Issues in a Resource Limited Environment

A myriad of potential ethical situations could arise for neurologists (26). Accounts of the Lombardy region of Italy detail harrowing decisions of life and death by ICU physicians (3). How do we weigh diseases such as Alzheimer's or Parkinson's against ventilated patients when asked about “life prognosis” or “prospective instrumental value to others” (4)? How do we factor in neurologic comorbidities when making triage decisions? While we hope to never reach this point, we must prepare for it. We must not categorically exclude those with chronic neurologic and cognitive disability. It is imperative we proactively discuss goals of care with patients outside the hospital to shield the frail from medical intervention that may provide potential harm. Now, is a time to develop a robust palliative care program for patients with limitation of therapeutic effort (LTE). Furthermore, these difficult ICU decisions should use advanced directives and living wills and be guided in a multidisciplinary fashion with ethical committees.

In the first weeks of the pandemic, we noticed many subtle clinical situations that already challenge our previous framework of clinical practice. A seemingly simple example is the extent of observation and work up in a transient ischemic attack. Does a patient on therapeutic anticoagulation and a low ABCD score for transient numbness warrant admission? Previously in our academic tertiary hospital, we would admit this patient and pursue an extensive stroke work up. Currently, the risk of exposure to COVID-19 in the hospital leads providers and patients to prefer outpatient workup, forgoing, or curtailing inpatient monitoring. How this impacts patient outcome is not certain. On the other hand, the risk of nosocomial infection previously existed, and the potential for harm was present in healthcare before COVID-19. How much higher this risk is now with COVID-19 is unexplored. These questions may lead to a fundamental risk assessment going forward where the marginal benefit of improved outcome for inpatient admission is weighed against the increased risks associated with hospital stay and procedures (27).

Our actions as specialists do not exist in a vacuum. We should note the impact our testing has on nurses and ancillary staff. For instance, we were consulted for abnormal neck movements in a prone-position COVID-19-positive patient. Our initial impulse was to order a 24-h EEG to capture this event. But a number of questions arose. What is the benefit of a 32-lead EEG established by an EEG technologist over a portable and limited EEG that can be established by a bedside provider who already had used PPE and was at the bedside? How does our diagnostic plan differ from pre-pandemic? What are the effects on patient outcome if we adjust our diagnostic and treatment algorithm in the setting of the COVID-19 pandemic? Our department is developing a collaborative protocol posed from these clinical questions.

Finally, how do we manage outpatients with progressive neurologic disease—the ones with limited life expectancy but who not ill enough to be in the hospital? An example is a man with longstanding amyotrophic lateral sclerosis (ALS) who is scheduled for outpatient gastrostomy tube placement. The interventional radiology team inquires if gastrostomy tube placement can be delayed as the healthcare system reduces use of equipment and staff for elective procedures. A fully informed discussion in a controlled setting with the patient and his family regarding the goals of care is important. We are still not sure how these discussions will be framed by the current crisis or used for triage, but we as neurologists are well-equipped for these discussions and should be proactive.

## Conclusion

COVID-19 has disrupted the neurologic healthcare ecosystem in the inpatient, outpatient, and research setting. It is paramount that we aid in preserving limited hospital resources and protect our patients and teams by critically assessing all clinical practices. What emerges are striking changes in clinical workflow and a chance to develop telemedicine and potentially difficult clinical-ethical decisions. Moving forward, we should be diligent in data collection and strive to understand how these workflow changes impact our patients. The silver lining in this pandemic is we have the opportunity as a specialty to revisit our practices and change for the better.

## Author Contributions

PC: study concept and design, acquisition of data, analysis and interpretation, critical revision of the manuscript for important intellectual content. TH: analysis and interpretation, critical revision of the manuscript for important intellectual content, study supervision. All authors read and approved the final manuscript.

## Conflict of Interest

The authors declare that the research was conducted in the absence of any commercial or financial relationships that could be construed as a potential conflict of interest.
